# Comparative Effectiveness of Moderna, Pfizer-BioNTech, and Janssen (Johnson & Johnson) Vaccines in Preventing COVID-19 Hospitalizations Among Adults Without Immunocompromising Conditions — United States, March–August 2021

**DOI:** 10.15585/mmwr.mm7038e1

**Published:** 2021-09-24

**Authors:** Wesley H. Self, Mark W. Tenforde, Jillian P. Rhoads, Manjusha Gaglani, Adit A. Ginde, David J. Douin, Samantha M. Olson, H. Keipp Talbot, Jonathan D. Casey, Nicholas M. Mohr, Anne Zepeski, Tresa McNeal, Shekhar Ghamande, Kevin W. Gibbs, D. Clark Files, David N. Hager, Arber Shehu, Matthew E. Prekker, Heidi L. Erickson, Michelle N. Gong, Amira Mohamed, Daniel J. Henning, Jay S. Steingrub, Ithan D. Peltan, Samuel M. Brown, Emily T. Martin, Arnold S. Monto, Akram Khan, Catherine L. Hough, Laurence W. Busse, Caitlin C. ten Lohuis, Abhijit Duggal, Jennifer G. Wilson, Alexandra June Gordon, Nida Qadir, Steven Y. Chang, Christopher Mallow, Carolina Rivas, Hilary M. Babcock, Jennie H. Kwon, Matthew C. Exline, Natasha Halasa, James D. Chappell, Adam S. Lauring, Carlos G. Grijalva, Todd W. Rice, Ian D. Jones, William B. Stubblefield, Adrienne Baughman, Kelsey N. Womack, Christopher J. Lindsell, Kimberly W. Hart, Yuwei Zhu, Lisa Mills, Sandra N. Lester, Megan M. Stumpf, Eric A. Naioti, Miwako Kobayashi, Jennifer R. Verani, Natalie J. Thornburg, Manish M. Patel, IVY Network, IVY Network, Nicole Calhoun, Kempapura Murthy, Judy Herrick, Amanda McKillop, Eric Hoffman, Martha Zayed, Michael Smith, Natalie Seattle, Jason Ettlinger, Elisa Priest, Jennifer Thomas, Alejandro Arroliga, Madhava Beeram, Ryan Kindle, Lori-Ann Kozikowski, Lesley De Souza, Scott Ouellette, Sherell Thornton-Thompson, Omar Mehkri, Kiran Ashok, Susan Gole, Alexander King, Bryan Poynter, Nicholas Stanley, Audrey Hendrickson, Ellen Maruggi, Tyler Scharber, Jeffrey Jorgensen, Robert Bowers, Jennifer King, Valerie Aston, Brent Armbruster, Richard E. Rothman, Rahul Nair, Jen-Ting (Tina) Chen, Sarah Karow, Emily Robart, Paulo Nunes Maldonado, Maryiam Khan, Preston So, Joe Levitt, Cynthia Perez, Anita Visweswaran, Jonasel Roque, Adreanne Rivera, Los Angeles, Trevor Frankel, Los Angeles, Jennifer Goff, David Huynh, Michelle Howell, Jennifer Friedel, Michael Tozier, Conner Driver, Michael Carricato, Alexandra Foster, Paul Nassar, Lori Stout, Zita Sibenaller, Alicia Walter, Jasmine Mares, Logan Olson, Bradley Clinansmith, Carolina Rivas, Hayley Gershengorn, EJ McSpadden, Rachel Truscon, Anne Kaniclides, Lara Thomas, Ramsay Bielak, Weronika Damek Valvano, Rebecca Fong, William J. Fitzsimmons, Christopher Blair, Andrew L. Valesano, Julie Gilbert, Christine D. Crider, Kyle A. Steinbock, Thomas C. Paulson, Layla A. Anderson, Christy Kampe, Jakea Johnson, Rendie McHenry, Marcia Blair, Douglas Conway, Mary LaRose, Leigha Landreth, Madeline Hicks, Lisa Parks, Jahnavi Bongu, David McDonald, Candice Cass, Sondra Seiler, David Park, Tiffany Hink, Meghan Wallace, Carey-Ann Burnham, Olivia G. Arter

**Affiliations:** ^1^Vanderbilt University Medical Center, Nashville, Tennessee; ^2^CDC COVID-19 Response Team; ^3^Baylor Scott & White Health, Temple, Texas; ^4^Texas A&M University College of Medicine, Temple, Texas; ^5^University of Colorado School of Medicine, Aurora, Colorado; ^6^University of Iowa, Iowa City, Iowa; ^7^Wake Forest University Baptist Medical Center, Winston-Salem, North Carolina; ^8^Johns Hopkins Hospital, Baltimore, Maryland; ^9^Hennepin County Medical Center, Minneapolis, Minnesota; ^10^Montefiore Healthcare Center, Albert Einstein College of Medicine, Bronx, New York; ^11^University of Washington School of Medicine, Seattle, Washington; ^12^Baystate Medical Center, Springfield, Massachusetts; ^13^Intermountain Medical Center and University of Utah, Salt Lake City, Utah; ^14^University of Michigan School of Public Health, Ann Arbor, Michigan; ^15^Oregon Health & Science University Hospital, Portland, Oregon; ^16^Emory University School of Medicine, Atlanta, Georgia; ^17^Cleveland Clinic, Cleveland, Ohio; ^18^Stanford University School of Medicine, Stanford, California; ^19^Ronald Reagan-UCLA Medical Center, Los Angeles, California; ^20^University of Miami, Miami, Florida; ^21^Washington University, St. Louis, Missouri; ^22^Ohio State University Wexner Medical Center, Columbus, Ohio; ^23^University of Michigan School of Medicine, Ann Arbor, Michigan.; Baylor Scott & White Health; Baylor Scott & White Health; Baylor Scott & White Health; Baylor Scott & White Health; Baylor Scott & White Health; Baylor Scott & White Health; Baylor Scott & White Health; Baylor Scott & White Health; Baylor Scott & White Health; Baylor Scott & White Health; Baylor Scott & White Health; Baylor Scott & White Health; Baylor Scott & White Health; Baystate Medical Center; Baystate Medical Center; Baystate Medical Center; Baystate Medical Center; Baystate Medical Center; Cleveland Clinic; Cleveland Clinic; Cleveland Clinic; Cleveland Clinic; Cleveland Clinic; Emory University; Hennepin County Medical Center; Hennepin County Medical Center; Hennepin County Medical Center; Intermountain Medical Center; Intermountain Medical Center; Intermountain Medical Center; Intermountain Medical Center; Intermountain Medical Center; Johns Hopkins University; Montefiore Medical Center; Montefiore Medical Center; Ohio State University; Ohio State University; Ohio State University; Ohio State University; Ohio State University; Stanford University; Stanford University; Stanford University; Stanford University; University of California; University of California; UCHealth University of Colorado Hospital; UCHealth University of Colorado Hospital; UCHealth University of Colorado Hospital; UCHealth University of Colorado Hospital; UCHealth University of Colorado Hospital; UCHealth University of Colorado Hospital; UCHealth University of Colorado Hospital; UCHealth University of Colorado Hospital; University of Iowa; University of Iowa; University of Iowa; University of Iowa; University of Iowa; University of Iowa; University of Iowa; University of Miami; University of Miami; University of Michigan; University of Michigan; University of Michigan; University of Michigan; University of Michigan; University of Michigan; University of Michigan; University of Michigan; University of Michigan; University of Michigan; University of Michigan; University of Washington; University of Washington; University of Washington; University of Washington; Vanderbilt University Medical Center; Vanderbilt University Medical Center; Vanderbilt University Medical Center; Vanderbilt University Medical Center; Vanderbilt University Medical Center; Wake Forest University; Wake Forest University; Wake Forest University; Wake Forest University; Washington University; Washington University; Washington University; Washington University; Washington University; Washington University; Washington University; Washington University; Washington University.

Three COVID-19 vaccines are authorized or approved for use among adults in the United States (*1*,*2*). Two 2-dose mRNA vaccines, mRNA-1273 from Moderna and BNT162b2 from Pfizer-BioNTech, received Emergency Use Authorization (EUA) by the Food and Drug Administration (FDA) in December 2020 for persons aged ≥18 years and aged ≥16 years, respectively. A 1-dose viral vector vaccine (Ad26.COV2 from Janssen [Johnson & Johnson]) received EUA in February 2021 for persons aged ≥18 years (*3*). The Pfizer-BioNTech vaccine received FDA approval for persons aged ≥16 years on August 23, 2021 (*4*). Current guidelines from FDA and CDC recommend vaccination of eligible persons with one of these three products, without preference for any specific vaccine (*4*,*5*). To assess vaccine effectiveness (VE) of these three products in preventing COVID-19 hospitalization, CDC and collaborators conducted a case-control analysis among 3,689 adults aged ≥18 years who were hospitalized at 21 U.S. hospitals across 18 states during March 11–August 15, 2021. An additional analysis compared serum antibody levels (anti-spike immunoglobulin G [IgG] and anti–receptor binding domain [RBD] IgG) to SARS-CoV-2, the virus that causes COVID-19, among 100 healthy volunteers enrolled at three hospitals 2–6 weeks after full vaccination with the Moderna, Pfizer-BioNTech, or Janssen COVID-19 vaccine. Patients with immunocompromising conditions were excluded. VE against COVID-19 hospitalizations was higher for the Moderna vaccine (93%; 95% confidence interval [CI] = 91%–95%) than for the Pfizer-BioNTech vaccine (88%; 95% CI = 85%–91%) (p = 0.011); VE for both mRNA vaccines was higher than that for the Janssen vaccine (71%; 95% CI = 56%–81%) (all p<0.001). Protection for the Pfizer-BioNTech vaccine declined 4 months after vaccination. Postvaccination anti-spike IgG and anti-RBD IgG levels were significantly lower in persons vaccinated with the Janssen vaccine than the Moderna or Pfizer-BioNTech vaccines. Although these real-world data suggest some variation in levels of protection by vaccine, all FDA-approved or authorized COVID-19 vaccines provide substantial protection against COVID-19 hospitalization.

For the VE analysis, adults aged ≥18 years without an immunocompromising condition admitted to 21 hospitals within the Influenza and Other Viruses in the Acutely Ill (IVY) Network were prospectively recruited for a case-control analysis (*6*,*7*). Case-patients were admitted to a hospital with COVID-19–like illness^†^ and a positive SARS-CoV-2 reverse transcription–polymerase chain reaction (RT-PCR) or antigen test result. Control-patients were adults admitted to a hospital^§^ who received a negative SARS-CoV-2 RT-PCR test result.

Patients or their proxies were interviewed to obtain information about demographic characteristics, clinical history, and COVID-19 vaccination.^¶^ Information regarding vaccine product received by patients was collected by interview and source verification (e.g., state vaccination registries, hospital electronic medical records, and pharmacy records), including vaccine lot numbers, when these were documented. A patient was considered fully vaccinated if the final vaccine dose (second dose for Moderna and Pfizer-BioNTech and the single Janssen dose) was received ≥14 days before illness onset.** Patients were excluded if they received a COVID-19 vaccine other than Moderna, Pfizer-BioNTech, or Janssen; received ≥1 vaccine dose but did not meet criteria for full vaccination; or received doses of two different COVID-19 vaccine products.

For the postvaccination antibody analysis, healthy adults aged ≥18 years with no known prior SARS-CoV-2 infection were recruited during April 6–June 4, 2021, at three IVY sites. Blood was collected 2–6 weeks after receipt of the second Moderna and Pfizer-BioNTech vaccine dose or the single Janssen vaccine dose. Sera were shipped to CDC, where they underwent testing for IgG against three SARS-CoV-2 antigens: the spike protein (anti-spike); the spike RBD (anti-RBD); and nucleocapsid (anti-nucleocapsid). IgG levels were measured using the V-PLEX SARS-CoV-2 panel 2 kit (Meso Scale Diagnostics) and reported in international binding antibody units (BAU) per milliliter. Two participants with anti-nucleocapsid antibodies (>11.8 BAU), which is suggestive of a prior SARS-CoV-2 infection, were excluded.

VE against COVID-19 hospitalization was estimated using logistic regression, comparing the odds of being fully vaccinated versus unvaccinated between case-patients and controls using the equation VE = 100 × (1 – adjusted odds ratio) (*6*,*7*). The regression model included an indicator variable for vaccine type (Moderna, Pfizer-BioNTech, or Janssen) and was adjusted for admission date, geographic region, age, sex, and race and Hispanic ethnicity. A separate model added an interaction term between product type and time since vaccination. Using these models, VE for mRNA vaccine products was estimated for the full surveillance period (March 11–August 15), as well as 14–120 days and >120 days after the receipt of the second dose. Because a limited number of patients received Janssen vaccine >120 days before illness onset (19 total), VE for the Janssen vaccine was not stratified by time. In addition to a VE estimate defining full vaccination as 14 days after a Janssen vaccine dose, a sensitivity analysis was conducted defining full vaccination as 28 days after a Janssen vaccine dose. Statistical differences by vaccine product and time were assessed based on p-values generated using the Tukey method for pair-wise multiple comparisons.

In the postvaccination antibody analysis, pairwise comparisons of the quantity of anti-spike IgG and anti-RBD IgG were made among participants, by vaccine product received, using the Wilcoxon rank-sum test. Analyses were conducted using R statistical software (version 4.0.3; R Foundation) and STATA (version 16; StataCorp). Procedures were approved as public health surveillance by each participating site and CDC^††^ and were conducted consistent with applicable federal law and CDC policy.^§§^

After excluding 1,786 patients from the VE analysis (936 for having an immunocompromising condition,^¶¶^ 566 who received ≥1 vaccine dose but were not fully vaccinated, and 284 who did not meet other eligibility criteria), 3,689 patients were included (1,682 case-patients and 2,007 control-patients). Overall, 2,362 (64.0%) patients were unvaccinated; 476 (12.9%) were fully vaccinated with the Moderna vaccine; 738 (20.0%) were fully vaccinated with the Pfizer-BioNTech vaccine; and 113 (3.1%) were fully vaccinated with the Janssen vaccine. Among all participants, median age was 58 years, 48% were female, 23% were non-Hispanic Black, and 18% were Hispanic (Table 1). VE against COVID-19 hospitalization during March 11–August 15, 2021, was higher for the Moderna vaccine (VE = 93%) than for the Pfizer-BioNTech vaccine (VE = 88%) (p = 0.011); VE for both mRNA vaccines was higher than that for the Janssen vaccine (VE = 71%) (all p<0.001) (Table 2). VE for the Moderna vaccine was 93% at 14–120 days (median = 66 days) after receipt of the second vaccine dose and 92% at >120 days (median = 141 days) (p = 1.000). VE for the Pfizer-BioNTech vaccine was 91% at 14–120 days (median = 69 days) after receipt of the second vaccine dose but declined significantly to 77% at >120 days (median = 143 days) (p<0.001).

**TABLE 1:** Characteristics of participants in the vaccine effectiveness analysis, including case-patients hospitalized with COVID-19 and control-patients hospitalized without COVID-19, by COVID-19 vaccine product received — 21 hospitals* in 18 U.S. states, March–August 2021

Characteristic^†^	No./Total no. (%)				
	All participants (N = 3,689)	Vaccine (fully vaccinated participants)^§^			Unvaccinated participants (n = 2,362)
		Moderna (n = 476)	Pfizer-BioNTech (n = 738)	Janssen (Johnson & Johnson) (n = 113)	
**COVID-19 case**	**1,682/3,689 (45.6)**	**54/476 (11.3)**	**128/738 (17.3)**	**37/113 (32.7)**	**1,463/2,362 (61.9)**
Median age (IQR, yrs)	58 (44–69)	66 (56–75)	68 (57–77)	61 (48–67)	53 (40–64)
**Age group, yrs**					
18–49	1,243/3,689 (33.7)	77/476 (16.2)	112/738 (15.2)	32/113 (28.3)	1,022/2,362 (43.3)
50–64	1,125/3,689 (30.5)	127/476 (26.7)	191/738 (25.9)	45/113 (39.8)	762/2,362 (32.3)
≥65	1,321/3,689 (35.8)	272/476 (57.1)	435/738 (58.9)	36/113 (31.9)	578/2,362 (24.5)
**Sex**					
Female	1,777/3,689 (48.2)	233/476 (49.0)	371/738 (50.3)	46/113 (40.7)	1,127/2,362 (47.7)
**Race/Ethnicity^¶^**					
White, non-Hispanic	1,960/3,689 (53.1)	291/476 (61.1)	480/738 (65.0)	58/113 (51.3)	1,131/2,362 (47.9)
Black, non-Hispanic	861/3,689 (23.3)	93/476 (19.5)	129/738 (17.5)	26/113 (23.0)	613/2,362 (26.0)
Any race, Hispanic	649/3,689 (17.6)	69/476 (14.5)	93/738 (12.6)	20/113 (17.7)	467/2,362 (19.8)
All other races, non-Hispanic	160/3,689 (4.3)	16/476 (3.4)	32/738 (4.3)	5/113 (4.4)	107/2,362 (4.5)
Unknown	59/3,689 (1.6)	7/476 (1.5)	4/738 (0.5)	4/113 (3.5)	44/2,362 (1.9)
U.S. Census region**					
Northeast	552/3,689 (15.0)	78/476 (16.4)	112/738 (15.2)	21/113 (18.6)	341/2,362 (14.4)
South	1,501/3,689 (40.7)	125/476 (26.3)	315/738 (42.7)	40/113 (35.4)	1,021/2,362 (43.2)
Midwest	836/3,689 (22.7)	155/476 (32.6)	166/738 (22.5)	27/113 (23.9)	488/2,362 (20.7)
West	800/3,689 (21.7)	118/476 (24.8)	145/738 (19.7)	25/113 (22.1)	512/2,362 (21.7)
**Residence in long-term care facility^††^**	155/3,557 (4.4)	29/463 (6.3)	68/712 (9.6)	4/111 (3.6)	54/2,271 (2.4)
**Has health insurance**	3,347/3,687 (90.8)	462/476 (97.1)	719/737 (97.6)	106/112 (94.6)	2,060/2,362 (87.2)
**Employed**	1,115/3,045 (36.6)	129/415 (31.1)	168/644 (26.1)	31/102 (30.4)	787/1,884 (41.8)
Health care worker	181/3,045 (5.9)	26/415 (6.3)	42/644 (6.5)	4/102 (3.9)	109/1,884 (5.8)
**Attended some college or more**	1,360/2,725 (49.9)	212/363 (58.4)	359/599 (59.9)	50/92 (54.4)	739/1,671 (44.2)
**≥1 hospital admission in past year**	1,380/3,434 (40.2)	233/456 (51.1)	325/701 (46.4)	52/109 (47.7)	770/2,168 (35.5)
**Underlying medical conditions^§§^**					
Chronic cardiovascular disease (including hypertension)	2201/3,688 (59.7)	341/475 (71.8)	567/738 (76.8)	75/113 (66.4)	1,218/2,362 (51.6)
Chronic lung disease	925/3688 (25.1)	145/475 (30.5)	224/738 (30.4)	35/113 (31.0)	521/2,362 (22.1)
Diabetes mellitus	1,091/3,688 (29.6)	173/475 (36.4)	267/738 (36.2)	33/113 (29.2)	618/2,362 (26.2)
Obesity by body mass index	1,829/3,648 (50.1)	203/474 (42.8)	335/733 (45.7)	53/113 (46.9)	1,238/2,328 (53.2)
**Self-reported prior laboratory-confirmed SARS-CoV-2 infection**	226/3,687 (6.1)	34/476 (7.1)	44/737 (6.0)	11/113 (9.7)	137/2,361 (5.8)
**Interval between second vaccine dose and symptom onset (or hospital admission for syndrome-negative control group), median no. of days (IQR)^¶¶^**	N/A	79 (46–112)	86 (51–119)	68 (36–111)	N/A

**Abbreviations:** IQR = interquartile range; N/A = not applicable.

* Hospitals by region included *Northeast*: Baystate Medical Center (Springfield, Massachusetts), Beth Israel Deaconess Medical Center (Boston, Massachusetts), Montefiore Medical Center (Bronx, New York); *South*: Vanderbilt University Medical Center (Nashville, Tennessee), University of Miami Medical Center (Miami, Florida), Emory University Medical Center (Atlanta, Georgia), Johns Hopkins Hospital (Baltimore, Maryland), Wake Forest University Baptist Medical Center (Winston-Salem, North Carolina), Baylor Scott & White Health (Temple, Texas); *Midwest*: University of Iowa Hospitals and Clinics (Iowa City, Iowa), University of Michigan Hospital (Ann Arbor, Michigan), Hennepin County Medical Center (Minneapolis, Minnesota), Barnes-Jewish Hospital (St. Louis, Missouri), Cleveland Clinic (Cleveland, Ohio), Ohio State University Wexner Medical Center (Columbus, Ohio); *West*: Stanford University Medical Center (Stanford, California), UCLA Medical Center (Los Angeles, California), UCHealth University of Colorado Hospital (Aurora, Colorado), Oregon Health & Science University Hospital (Portland, Oregon), Intermountain Medical Center (Murray, Utah), University of Washington (Seattle, Washington).

**^†^** Data are not complete for all characteristics in the table; denominators are included in the table indicating the number of patients with available data for each characteristic.

**^§^** Fully vaccinated with mRNA COVID-19 vaccines defined as ≥14 days from dose 2; fully vaccinated with Janssen (Johnson & Johnson) vaccine defined as ≥14 days from dose 1.

^¶^ Racial and ethnic groups were reported by the patient or proxy.

** *Northeast*: Connecticut, Maine, Massachusetts, New Hampshire, New Jersey, New York, Pennsylvania, Rhode Island, and Vermont; *Midwest*: Illinois, Indiana, Iowa, Kansas, Michigan, Minnesota, Missouri, Nebraska, North Dakota, Ohio, South Dakota, and Wisconsin; *South*: Alabama, Arkansas, Delaware, District of Columbia, Florida, Georgia, Kentucky, Louisiana, Maryland, Mississippi, North Carolina, Oklahoma, South Carolina, Tennessee, Texas, Virginia, and West Virginia; *West*: Alaska, Arizona, California, Colorado, Hawaii, Idaho, Montana, Nevada, New Mexico, Oregon, Utah, Washington, and Wyoming.

^††^ Long-term care facility included reporting living in a nursing home, assisted living home, or rehabilitation hospital or other subacute or chronic facility before the hospital admission.

^§§^ Underlying medical condition categories were obtained through medical chart review by trained personnel.

^¶¶^ Among fully vaccinated patients.

**TABLE 2:** COVID-19 vaccine effectiveness* against COVID-19–associated hospitalization among adults without immunocompromising conditions, by vaccine product — 21 hospitals in 18 U.S. states,^†^ March–August 2021

Vaccine/Period	Vaccinated patients/Total patients (%)		VE against COVID-19 hospitalization (95% CI)
	Case-patients	Control-patients	
**Moderna VE after full vaccination**			
Full surveillance period^§^	54/1,517 (3.6)	422/1,321 (31.9)	93 (91–95)
14–120 days after full vaccination	36/1,499 (2.4)	345/1,244 (27.7)	93 (90–95)
>120 days after full vaccination	18/1,481 (1.2)	77/976 (7.9)	92 (87–96)
**Pfizer-BioNTech VE after full vaccination**			
Full surveillance period	128/1,591 (8.0)	610/1,509 (40.4)	88 (85–91)
14–120 days after full vaccination	65/1,528 (4.3)	495/1,394 (35.5)	91 (88–93)
>120 days after full vaccination	63/1,526 (4.1)	115/1,014 (11.3)	77 (67–84)
**Janssen (Johnson & Johnson) VE after full vaccination**			
Full surveillance period	37/1,500 (2.5)	76/975 (7.8)	71 (56–81)
>28 days after full vaccination	33/1,496 (2.2)	59/958 (6.2)	68 (49–80)

**Abbreviations:** CI = confidence interval; VE = vaccine effectiveness.

* VE was estimated using logistic regression comparing the odds of being fully vaccinated with the Moderna, Pfizer-BioNTech or Janssen (Johnson & Johnson) COVID-19 vaccine versus being unvaccinated in case-patients and control-patients using the equation VE = 100 × (1 – odds ratio). Models were adjusted for date of hospital admission (biweekly intervals), U.S. Department of Health and Human Services region of hospital, age group (18–49, 50–64, ≥65 years), sex, and race/ethnicity (non-Hispanic White, non-Hispanic Black, Hispanic of any race, non-Hispanic Other, or unknown). Binary time since full vaccination was added to the model with results for 14–120 days and >120 days shown.

^†^ Hospitals by region included *Northeast*: Baystate Medical Center (Springfield, Massachusetts), Beth Israel Deaconess Medical Center (Boston, Massachusetts), Montefiore Medical Center (Bronx, New York); *South*: Vanderbilt University Medical Center (Nashville, Tennessee), University of Miami Medical Center (Miami, Florida), Emory University Medical Center (Atlanta, Georgia), Johns Hopkins Hospital (Baltimore, Maryland), Wake Forest University Baptist Medical Center (Winston-Salem, North Carolina), Baylor Scott & White Health (Temple, Texas); *Midwest*: University of Iowa Hospitals and Clinics (Iowa City, Iowa), University of Michigan Hospital (Ann Arbor, Michigan), Hennepin County Medical Center (Minneapolis, Minnesota), Barnes-Jewish Hospital (St. Louis, Missouri), Cleveland Clinic (Cleveland, Ohio), Ohio State University Wexner Medical Center (Columbus, Ohio); *West*: Stanford University Medical Center (Stanford, California), UCLA Medical Center (Los Angeles, California), UCHealth University of Colorado Hospital (Aurora, Colorado), Oregon Health & Science University Hospital (Portland, Oregon), Intermountain Medical Center (Murray, Utah), University of Washington (Seattle, Washington).

^§^ The full surveillance period included hospital admissions during March 11–August 15, 2021.

The postvaccination antibody analysis included 100 healthy volunteers, 32 fully vaccinated with Moderna (median age = 31 years; median interval from second vaccine dose to blood draw = 28 days), 51 fully vaccinated with Pfizer-BioNTech (median age = 27 years; median interval from second dose to blood draw = 27 days), and 17 fully vaccinated with Janssen (median age = 31 years; median interval from vaccine dose to blood draw = 35 days). Anti-RBD levels were higher in participants vaccinated with the Moderna vaccine (median = 4,333; interquartile range [IQR] = 3,134–7,197; geometric mean = 4,274; 95% CI = 3,393–5,384 BAU/mL) than in those who received the Pfizer-BioNTech vaccine (median = 3,217; IQR = 2,048–4,668; geometric mean = 2,950; 95% CI = 2,325–3,742 BAU/mL) (p = 0.033) or the Janssen vaccine (median = 57; IQR = 26–94; geometric mean = 51; 95% CI = 30–90 BAU/mL) (p<0.001) (Figure). Anti-spike IgG levels in participants vaccinated with the Moderna vaccine (median = 3,236; IQR = 2,125–4,975, geometric mean = 3,059; 95% CI = 2,479–3,774 BAU/mL) did not significantly differ from those in recipients of the Pfizer-BioNTech vaccine (median = 2,983; IQR = 1,954–4,059; geometric mean = 2,444; 95% CI = 1,936–3,085 BAU/mL) (p = 0.217), but were significantly higher than levels in participants who received the Janssen vaccine (median = 59; IQR = 30–104; geometric mean = 56; 95% CI = 32–97 BAU/mL) (p<0.001).

**FIGURE:**
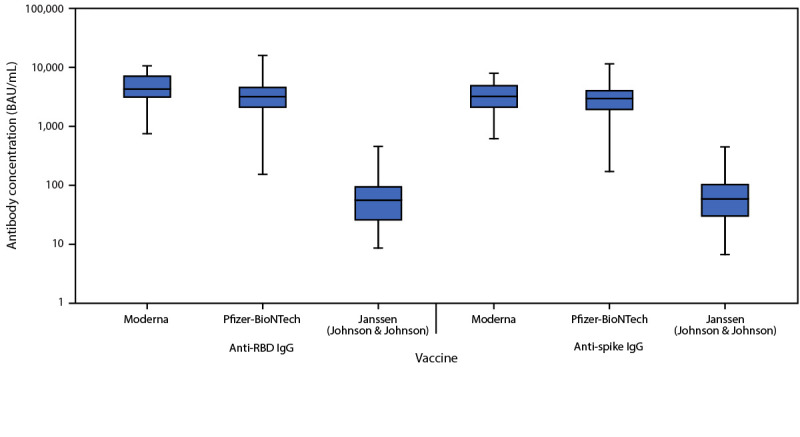
Serum anti–receptor binding domain and anti-spike immunoglobulin G levels 2–6 weeks after full vaccination among healthy adult volunteers — three hospitals in three U.S. states,*^,†^ April–June 2021 **Abbreviations:** BAU = binding antibody units; IgG = immunoglobulin G; IQR = interquartile range; RBD = receptor binding domain. * Anti-RBD and anti-spike IgG levels were measured in sera of healthy volunteers 2-6 weeks after a second dose of the Moderna or Pfizer-BioNTech COVID-19 vaccine and the first dose of the Janssen COVID-19 vaccine. In these box and whisker plots, the central horizontal line of each box plot represents the median, with the box denoting the IQR, and the whiskers representing the minimum and maximum values. Two volunteers with anti-nucleocapsid IgG antibodies, indicative of a prior SARS-CoV-2 infection, were excluded from this analysis. ^†^ Hospitals that recruited healthy adult volunteers included Beth Israel Deaconess Medical Center (Boston, Massachusetts), Vanderbilt University Medical Center (Nashville, Tennessee), and Wake Forest University Baptist Medical Center (Winston-Salem, North Carolina).

## Discussion

Two-dose regimens of the Moderna and Pfizer-BioNTech mRNA vaccines provided a high level of protection against COVID-19 hospitalizations in a real-world evaluation at 21 U.S. hospitals during March–August 2021. VE against COVID-19 hospitalization for Moderna and Pfizer-BioNTech vaccines was 93% and 88%, respectively, whereas the single-dose Janssen vaccine had somewhat lower VE at 71%. Persons vaccinated with Janssen vaccine also had lower postvaccination anti-SARS-CoV-2 antibody levels than did recipients of mRNA vaccines. Although an immunologic correlate of protection has not been established for COVID-19 vaccines, antibody titers after infection and vaccination have been associated with protection (*8*). These real-world data suggest that the 2-dose Moderna and Pfizer-BioNTech mRNA vaccine regimens provide more protection than does the 1-dose Janssen viral vector vaccine regimen. Although the Janssen vaccine had lower observed VE, 1 dose of Janssen vaccine still reduced risk for COVID-19–associated hospitalization by 71%.

VE against COVID-19 hospitalization was slightly lower for the 2-dose Pfizer-BioNTech vaccine than the Moderna vaccine, with this difference driven by a decline in VE after 120 days for the Pfizer-BioNTech but not the Moderna vaccine. The Moderna vaccine also produced higher postvaccination anti-RBD antibody levels than did the Pfizer-BioNTech vaccine. Differences in VE between the Moderna and Pfizer-BioNTech vaccine might be due to higher mRNA content in the Moderna vaccine, differences in timing between doses (3 weeks for Pfizer-BioNTech versus 4 weeks for Moderna), or possible differences between groups that received each vaccine that were not accounted for in the analysis (*9*).

The findings in this report are subject to at least six limitations. First, this analysis did not consider children, immunocompromised adults, or VE against COVID-19 that did not result in hospitalization. Second, the CIs for the Janssen VE estimates were wide because of the relatively small number of patients who received this vaccine. Third, follow-up time was limited to approximately 29 weeks since receipt of full vaccination, and further surveillance of VE over time is warranted. Fourth, although VE estimates were adjusted for relevant potential confounders, residual confounding is possible. Fifth, product-specific VE by variant, including against Delta variants (B.1.617.2 and AY sublineages), was not evaluated. Finally, antibody levels were measured at only a single time point 2–6 weeks after vaccination and changes in antibody response over time as well as cell-mediated immune responses were not assessed.

Two-dose series of the Moderna and Pfizer-BioNTech mRNA COVID-19 vaccines provided high VE for the prevention of COVID-19 hospitalizations during March–August 2021. Protection for the Pfizer-BioNTech vaccine declined 4 months after vaccination. A single dose of the Janssen viral vector vaccine had comparatively lower anti-SARS-CoV-2 antibody response and VE against COVID-19 hospitalizations. Understanding differences in VE by vaccine product can guide individual choices and policy recommendations regarding vaccine boosters. All FDA-approved or authorized COVID-19 vaccines provide substantial protection against COVID-19 hospitalization.

**Summary**

**What is already known about this topic?**

Two 2-dose mRNA COVID-19 vaccines (from Pfizer-BioNTech and Moderna) and a 1-dose viral vector vaccine (from Janssen [Johnson & Johnson]) are currently used in the United States.

**What is added by this report?**

Among U.S. adults without immunocompromising conditions, vaccine effectiveness against COVID-19 hospitalization during March 11–August 15, 2021, was higher for the Moderna vaccine (93%) than the Pfizer-BioNTech vaccine (88%) and the Janssen vaccine (71%).

**What are the implications for public health practice?**

Although these real-world data suggest some variation in levels of protection by vaccine, all FDA-approved or authorized COVID-19 vaccines provide substantial protection against COVID-19 hospitalization.

## Footnotes

* These authors contributed equally to this report.

† COVID-19–like illness was defined as having one or more of the following: fever, cough, shortness of breath, loss of taste, loss of sense of smell, use of respiratory support for the acute illness, or new pulmonary findings on chest imaging consistent with pneumonia.

§ Control-patients included test-negative controls (persons with COVID-19–like illness who received negative SARS-CoV-2 RT-PCR test results) and syndrome-negative controls (a second control group of persons without COVID-19–like illness who also received negative SARS-CoV-2 RT-PCR test results). VE estimates stratified by control groups were highly similar and control groups were combined for this analysis.

¶ Vaccine was considered to have been administered if vaccination dates and product names were verified through medical records, state immunization registries, vaccination record cards, or provider or pharmacy records, or if plausibly reported by patient or proxy with date and location of vaccination.

** The date of illness onset was used for cases and controls with COVID-19–like illness with median value imputed if missing. For controls without COVID-19–like illness, the date of admission minus the median number of days between illness onset and admission for patients with COVID-19 was used for a date of illness onset, also referred to as illness onset for this report.

†† All activities were approved by participating institutions as public health surveillance activities, except postvaccination blood collection that received institutional review board approval at a single site (Wake Forest University).

§§ 45 C.F.R. part 46.102(l)(2), 21 C.F.R. part 56; 42 U.S.C. Sect. 241(d); 5 U.S.C. Sect. 552a; 44 U.S.C. Sect. 3501 et seq.

¶¶ Immunocompromising conditions included having one or more of the following: active solid organ cancer (active cancer defined as treatment for the cancer or newly diagnosed cancer in the past 6 months), active hematologic cancer (such as leukemia, lymphoma, or myeloma), HIV infection without AIDS, AIDS, congenital immunodeficiency syndrome, prior splenectomy, prior solid organ transplant, immunosuppressive medication, systemic lupus erythematosus, rheumatoid arthritis, psoriasis, scleroderma, or inflammatory bowel disease including Crohn’s disease or ulcerative colitis.
